# Chicago Medical Society

**Published:** 1867-10

**Authors:** 


### Proceedings of Societies,

### CHICAGO MEDICAL SOCIETY.

### NEURALGIA PRODUCED BY A FATTY TUMOR.

Dr. Fisher exhibited a large fatty tumor, which he had removed from a male patient fifty years of age. The tumor, situated between the upper portion of the left scapula and spine, was first observed seven years ago. The growth increased very slowly in size, giving the patient scarcely any inconvenience, till about four years ago, when quite severe neuralgic pain was experienced in the left arm and side, and under the scapula. It was for this pain, especially, that the patient sought surgical aid.

The removal of the tumor, which was attended with considerable difficulty, in consequence of its very deep attachments with the facial and transverse spinal processes, relieved the pain.

Dr. Fisher reported the result of the *post mortem* examination of Dr. Orren Smith, whose death is recorded on another page.

Dr. Smith had suffered, in early life, from a severe attack of rheumatism, which resulted in serious cardiac disease, from which he suffered, more or less, to the day of his death.

The heart was hypertrophied and dilated, its texture soft, and the mitral and aortic valves thickened and insufficient. The pericardium was attached, throughout almost its whole extent, to the heart. There was extensive adhesions between the lungs and thoracic walls.

The liver was contracted, mottled, and uneven; the kidneys hard and contracted.

### FATTY DEGENERATION OF KIDNEYS IN PHTHISIS.

Dr. Fenn exhibited the kidneys of a female patient, forty years of age, who had died at the Cook County Hospital.

The patient had suffered from symptoms of phthisis for several years. During the later period of her life, she had suffered from disease of the kidneys. The urine was albuminous to a remarkable degree. The kidneys, exhibited, were typical specimens of fatty degeneration.

### RETINITIS ASSOCIATED WITH BRIGHT'S DISEASE.

Dr. Holmes reported the case of a young man, from the country, aged nineteen years, who consulted him in reference to dimness of vision, which he had experienced during convalescence from a severe illness. From the description of the patient, it appeared that the disease was typhoid fever. Vision was so impaired, that the patient could scarcely conduct himself with safety in the street. By simple inspection the eyes appeared perfectly normal. With the aid of the ophthalmoscope, the retina of each eye was shown to be in a condition of extreme congestion, being dark red, the vessels engorged, the optic nerve disk being absolutely obscured. There were a very few points of blood extravasated from the capillaries. The urine was found to be perfectly transparent, like pure water. Both nitric acid and heat precipitated a very large amount of albumen, causing the urine to look like milk. There was little derangement of the heart's action. The patient died soon after returning home.

Dr. Holmes referred to four other cases of retinitis, complicating Bright's disease, which he had before reported to the Society, and in which the ophthalmoscopic appearances were so characteristic, that the diagnosis of Bright's disease was at once declared.

The subsequent examination of the urine and the course of the disease verified the diagnosis.

In each of these cases there were the typical appearances, described in our recent works, and delineated in the best collections of ophthalmoscopic plates.

"These appearances consist of a swelling of the optic nerve disk, which is encircled by an opaque gray zone; this in turn is surrounded by a white belt, radiating lines of yellowish white spots being observed, external to the optic nerve, and minute patches of extravasated blood being scattered in different portions of the retina."

In a majority of cases, a portion only of these symptoms are found together.

The appearances described in the case, reported this evening, are perhaps more liable to be found in the early than in the later stages of the retinitis.

Dr. Hildreth stated that the fact, that similar appearances were observed in diabetes, might lead to error in diagnosis by means of the ophthalmoscope alone.

### INTRAOCULAR TUMOR.

Dr. Holmes exhibited a singular intraocular tumor which he had removed from a male patient, forty years of age, at the Chicago Charitable Eye and Ear Infirmary. He had experienced, for two years, quite extensive dimness of vision in the right eye without any known cause, and without change in the appearance of the eye.

Suddenly, in the early part of July last, he was seized with a most violent pain in the eye, which was so persistent as to resist the skill of his physician and impair his general health.

On entering the Infirmary, a few days since, the eye was found soft, on palpation, exceedingly painful and tender, the anterior chamber being filled with blood. The diagnosis was uncertain.

Extirpation of the globe was considered necessary, not only to relieve the distress of the patient, but also to prevent disease of the other eye.

At the lower and nasal side of the fundus, was a smooth and spherical tumor, about a fourth of an inch in diameter. At the lower portion of the fundus was the chrystalline lens, opaque, but round in size, and partially involved in the substance of the tumor. The lens had evidently become spontaneously dislocated, in consequence of the atrophy of the zonula of Zinn, and the dissolution of the vitreous humor.

The iris and ciliary bodies were so atrophied, that the pupil was equal in size to the cornea.

The space between the cornea and tumor was filled with a thick bloody fluid.

The retina and choroid were partially detached from each other, the latter being also detached from the scloretic.

Dr. Lyman describes the microscopic character of the tumor, as follows:—

The cells are of an oval shape, pretty uniformly resembling the pus-cell, in size and superficial aspect, but not presenting the distinct nuclei of the pus-cell, when treated with acetic acid.

There are numerous minute fat globules, and plates of cholesterine.

